# Pulmonary thrombotic pulmonary hypertension managed using antithrombotic and pulmonary vasodilator treatment

**DOI:** 10.1111/jvim.17089

**Published:** 2024-04-25

**Authors:** Rina Horikawa, Ryohei Suzuki, Yunosuke Yuchi, Shuji Satomi, Takahiro Saito, Takahiro Teshima, Hirotaka Matsumoto

**Affiliations:** ^1^ Laboratory of Veterinary Internal Medicine, School of Veterinary Science, Faculty of Veterinary Medicine, Nippon Veterinary and Life Science University Tokyo Japan; ^2^ Takahashi Animal Hospital Saitama Japan

**Keywords:** beraprost sodium, contrast‐enhanced computed tomography, monteplase, myocardial strain, pulmonary vascular resistance, speckle tracking echocardiography

## Abstract

An 8‐year‐old Leonberger receiving immunosuppressive treatment with clinical signs of acute dyspnea, cyanosis, and difficulty standing was referred to our institution (Day 1). Treatment including oxygen, clopidogrel, and low‐molecular‐weight heparin was initiated for suspected pulmonary thrombosis. However, exertional dyspnea persisted until Day 10, and increased tricuspid regurgitation velocity, pulmonary vascular resistance, and McConnell's signs also were observed. Thus, beraprost sodium was administered PO on Day 11 to treat suspected pulmonary hypertension. On Day 13, contrast‐enhanced computed tomography identified extensive contrast defects in the pulmonary arteries, and IV monteplase was administered on Days 14 and 18, with marked improvement in respiratory status and exertional dyspnea on Day 20. Right ventricular function and McConnell signs also improved, and tricuspid regurgitation velocity and pulmonary vascular resistance decreased. On Day 250, echocardiography indicated further improvement in pulmonary hypertension pathophysiology. The patient was still progressing well with antithrombotic and pulmonary vasodilator treatment 400 days later.

**Abbreviations**
2D‐STE: 2‐dimensional speckle‐tracking echocardiography
BPS: beraprost sodium
CT: computed tomography
FDP: fibrinogen‐fibrin degradation product
LV: left ventricular
LV‐SC: left ventricular circumferential directions
LV‐SL: left ventricular longitudinal directions
PH: pulmonary hypertension
PVR: pulmonary vascular resistance
PVRecho: pulmonary vascular resistance estimated by echocardiography
RI: reference interval
RV FACn: RV fractional area change normalized by body weight
RV: right ventricular
RV‐SL: RV longitudinal direction of the RV free wall
RVSVI: RV stroke volume normalized by body surface area
TAPSE: tricuspid annular plane systolic excursion
TAPSEn: tricuspid annular plane systolic excursion normalized by body weight
TR: tricuspid value regurgitation

## 1 INTRODUCTION

Pulmonary hypertension (PH) is defined as abnormally increased pressure within the pulmonary vasculature, which can result from increased pulmonary blood flow, increased pulmonary vascular resistance, increased pulmonary venous pressure, or a combination of these mechanisms.^1^ This abnormal hemodynamic state occurs primarily in the pulmonary artery or secondary to various other diseases that are classified into 6 groups according to the consensus guidelines of the American College of Veterinary Internal Medicine, namely: Group 1, pulmonary arterial hypertension; Group 2, left‐sided heart disease; Group 3, respiratory disease/hypoxia; Group 4, pulmonary emboli/pulmonary thrombi/pulmonary thromboemboli; Group 5, parasitic disease; and Group 6, disorders that are multifactorial or with unclear mechanisms. Pulmonary thrombosis (Group 4) is a potentially fatal disease that presents with acute respiratory signs. Guidelines for the treatment of pulmonary thrombosis in humans recommend the use of antithrombotic treatments, including thrombolytics, anticoagulants, and antiplatelet agents.^1^, ^2^ Additionally, the use of pulmonary vasodilators may be considered when pulmonary circulation does not improve sufficiently after administration of the aforementioned treatments.^2^ In humans, several pulmonary vasodilators have proved effective for treating PH, including phosphodiesterase‐5 inhibitors (eg, sildenafil), endothelin receptor inhibitors, and prostacyclin analogs (beraprost sodium [BPS]). Conversely, only a few studies have been reported on pulmonary thrombosis in dogs,^3^, ^4^, ^5^ and none has described using pulmonary vasodilators for its management. A previous study in dogs has reported a dosage of 15 μg/kg BPS as a treatment for PH, which dilates both the pulmonary and systemic blood vessels and improves circulation.^6^ No previous studies have reported treatment of PH using BPS in dogs with acute pulmonary thrombosis. We report a dog with PH caused by pulmonary thrombosis that was well controlled in the acute and chronic phases (approximately 400 days) with antithrombotic and pulmonary vasodilator treatment, including BPS.

## 2 CASE PRESENTATION

An 8‐year‐old intact female Leonberger weighing 54 kg was referred with chief complaints of acute dyspnea, cyanosis, and difficulty standing (Day 1). The dog had been receiving immunosuppressants (prednisolone [0.6 mg/kg PO q24h] and cyclosporine [5.5 mg/kg PO q24h]) for >1 year for treatment of immune‐mediated arthritis. The dog occasionally experienced mild lameness, which generally was controlled with PO medication. Additionally, the dog had dermatophytosis which was being treated with itraconazole (7 mg/kg PO q24h). Furthermore, it also was receiving heartworm (*Dirofilaria immitis*) prophylaxis with milbemycin (0.5 mg/kg PO q30d). Clinical signs at presentation differed from the initial report and included acute respiratory distress, cyanosis, and difficulty standing. Physical examination disclosed hyperthermia (39.3°C), tachycardia (172 bpm), and acute dyspnea. Mucous membranes were pale and percutaneous arterial oxygen saturation at presentation was 90%. The dog was immediately hospitalized in a 40% oxygen cage, where peripheral oxygen saturation increased to 94%‐96%. Auscultation did not disclose any heart murmurs.

**FIGURE 1:**
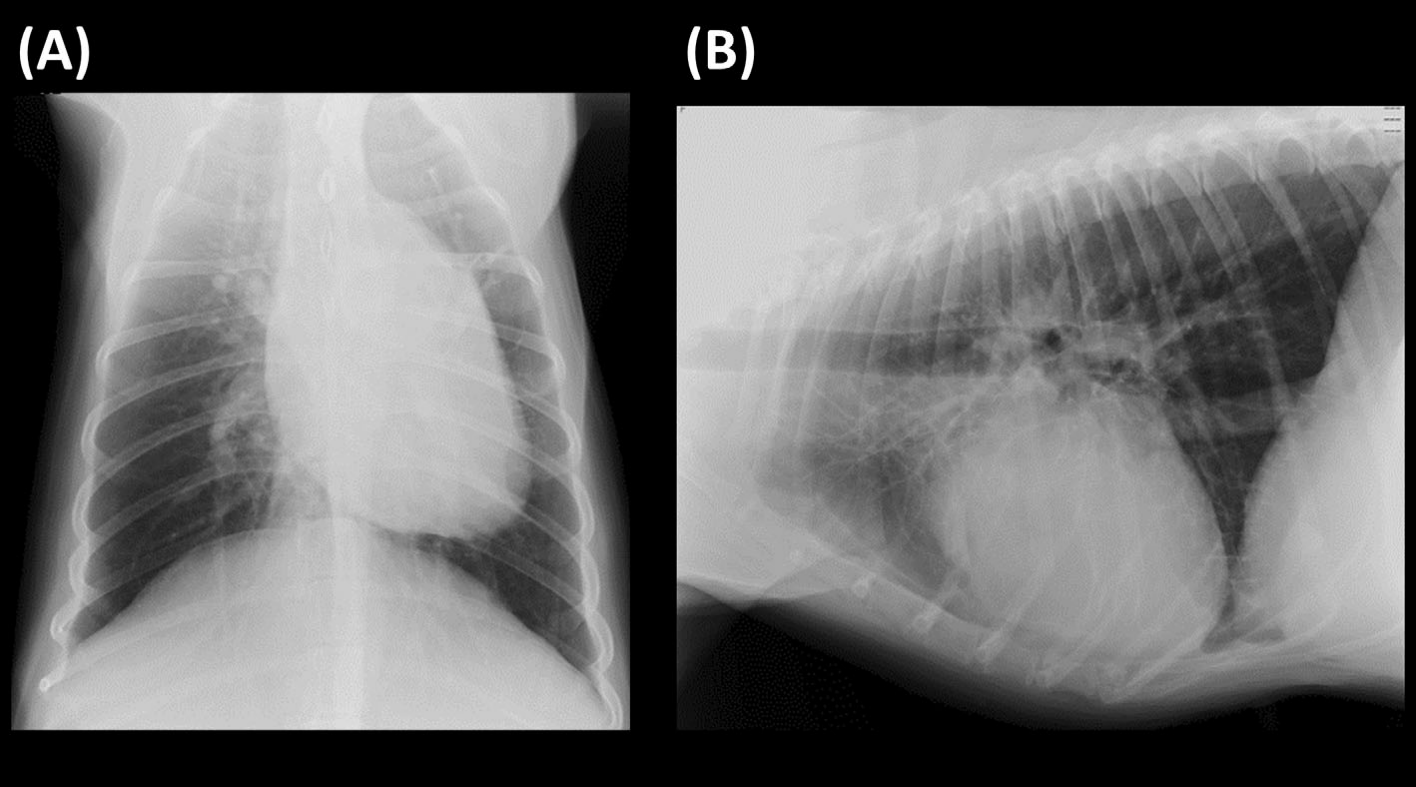
Transthoracic radiography on Day 1. Left figure represents the dorsoventral views (A), and right figure represents right lateral views (B). On Day 1, mild enlargement of the right pulmonary artery is observed. However, there were no significant abnormalities in the lung parenchyma that could have caused respiratory distress.

Electrocardiography and non‐invasive blood pressure measurements identified no abnormalities. A CBC showed no clinically relevant abnormalities, except for mildly increased white blood cell count (14 400/μL). Serum biochemistry identified increased activities of the following liver enzymes: alkaline phosphatase (5050 U/L; reference interval [RI], 47‐254 U/L), alanine transaminase (189 U/L; RI, 14‐68 U/L), and gamma‐glutamyl transpeptidase (22 U/L; RI, 2‐15 U/L) without any ultrasonographic abnormalities in the liver, suggesting prednisolone‐induced hepatic injury. No abnormalities of kidney function were observed including blood urea nitrogen (14.7 mg/dL; RI, 9.2‐29.2 mg/dL) and serum creatinine (0.6 mg/dL; RI, 0.4‐1.4 mg/dL) concentrations. Hypoalbuminemia (1.5 mg/dL; RI, 2.0‐3.2 μg/mL) and increased C‐reactive protein concentration (6.9 μg/mL; RI, <1.0 μg/mL) also were observed. A urine dipstick test which was performed previously was negative for protein. Coagulation tests showed abnormally high concentrations of fibrinogen‐fibrin degradation products (FDP; 28.4 μg/mL; RI, 0.0‐4.0 μg/mL), d‐dimer (16.1 μg/mL; RI, 0.0‐2.0 μg/mL), and fibrinogen (644 mg/dL; RI, 150‐350 mg/dL). No abnormalities were observed in prothrombin time (7.4 s; RI, 6.0‐9.5 s), activated partial thromboplastin time (16.5 s; RI, 9.0‐16.5 s), and antithrombin III (112.9%; RI, 70%‐160%). Venous blood gas analysis identified no abnormalities other than metabolic acidemia, mainly associated with a high concentration of lactate (pH 7.26; RI, 7.35‐7.45), carbon dioxide partial pressure (42 mmHg; RI, 35‐45 mmHg), bicarbonate ion concentration (18.8 mmol/L; RI, 22‐26 mmol/L), and lactate concentration (9.2 mmol/L; RI, 0.3‐2.5 mmol/L). Thoracic radiography indicated an enlarged right heart and pulmonary artery (Figure 1). Vertebral heart size was 11.8 vertebrae and no radiographic findings indicating pulmonary infiltration were observed. Conventional, 2‐dimensional, and Doppler echocardiography were performed using Vivid E95 echocardiographic system (GE Healthcare, Tokyo, Japan). All echocardiographic measurements were performed using an offline workstation (EchoPAC, version 204; GE Healthcare, Tokyo, Japan). Echocardiography performed by the first veterinarian indicated subjective dilatation of the right ventricle (RV) and right atrium and moderate flattening of the interventricular septum.^7^ Measured echocardiographic variables are summarized in Supplementary Table 1. Retrospective analysis of the data indicated RV and right atrial enlargement.^8^ The tricuspid regurgitation (TR) velocity was 3.4 m/s. Although the dog was diagnosed with a high probability of PH according to the AVCIM guidelines,^1^ a definitive cause could not be identified. The first veterinarian who examined the dog suspected respiratory failure associated with acute pulmonary thrombosis. The dog was rested in an oxygen cage and clopidogrel (1 mg/kg PO q24h), low‐molecular‐weight heparin (18.8 U/kg/h continuous rate infusion), and ampicillin (20 mg/kg IV q12h, because of the possibility of infection) were administered. After treatment, respiratory conditions stabilized in oxygen but exertional dyspnea persisted.

On Day 10, the dog's respiratory status worsened, and it was transferred to our cardiology department. Respiratory rate in 40% oxygen room was 52 per minute and cyanosis persisted after excitation, but pulmonary auscultation identified no obvious abnormalities. Systemic blood pressure was slightly increased (average systolic systemic blood pressure, 157 mmHg). Blood coagulation tests showed that the activated partial thromboplastin time (17.7 s) and fibrinogen concentration (>999 mg/dL) were higher than observed on Day 1. On Day 10, FDP and d‐dimer concentrations decreased to 4.7 and 2.4 μg/mL, respectively.

**FIGURE 2:**
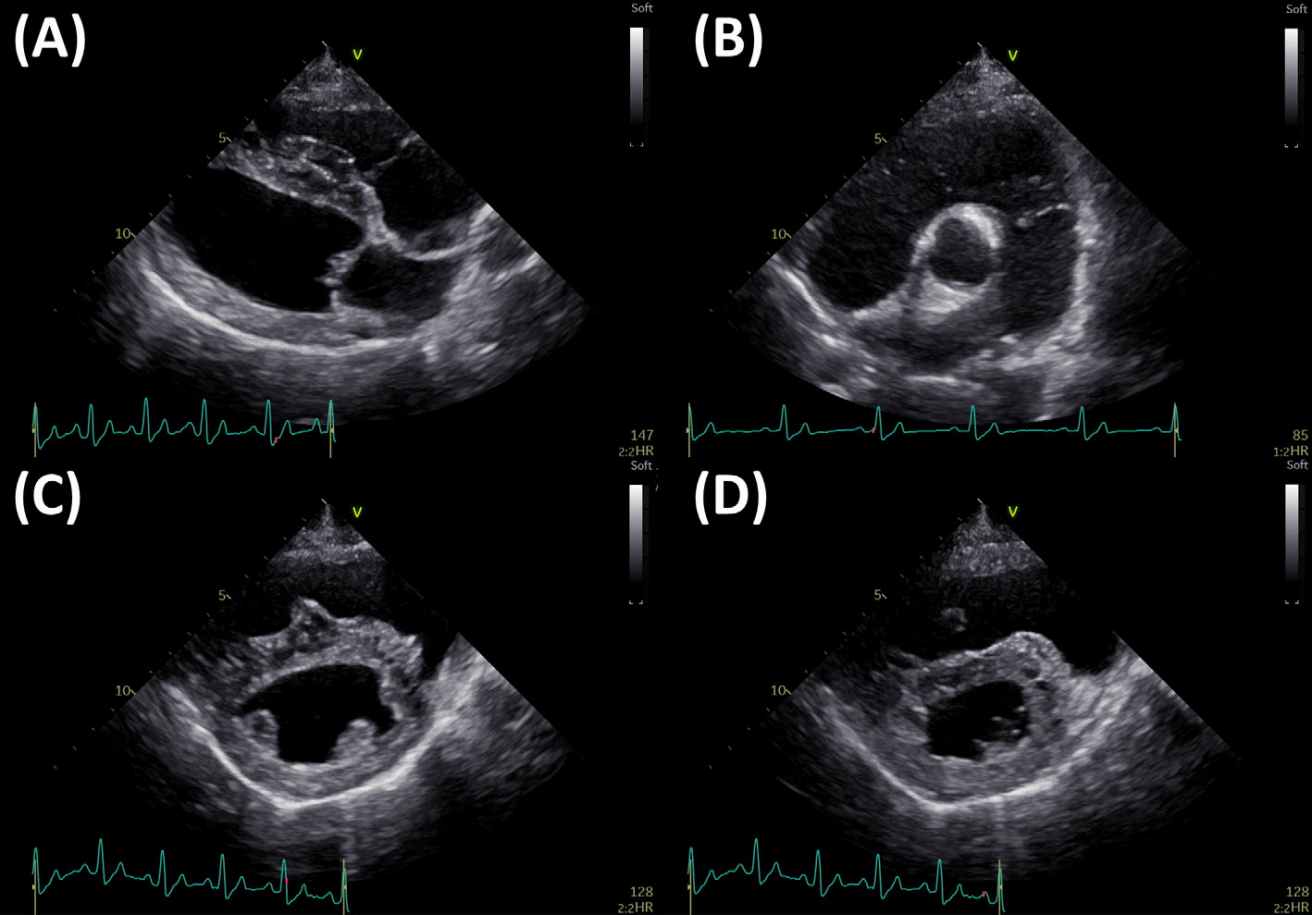
Echocardiographic views on Day 10. Right parasternal long‐axis view (A), right parasternal short‐axis view at the level of pulmonary artery (B), right parasternal short‐axis view at the level of papillary muscle (C: end‐diastole; D: end‐systole). Echocardiography revealed dilatation of the right heart and pulmonary arteries. In addition, flattening of the interventricular septum and narrowing of the left ventricle were observed.

**FIGURE 3:**
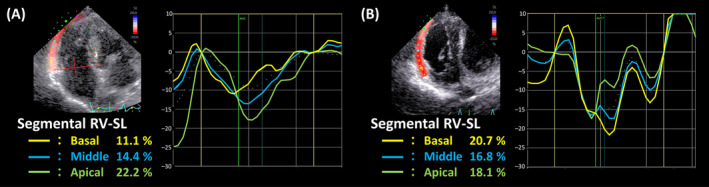
Longitudinal myocardial strains of the segmental right ventricular free wall (RV‐SL) on Days 10 (A) and 20 (B). On Day 10, the basal and middle RV‐SL were low but the apical RV‐SL was preserved, indicating McConnell's signs. In contrast, basal and middle RV‐SL increased on Day 20.

**FIGURE 4:**
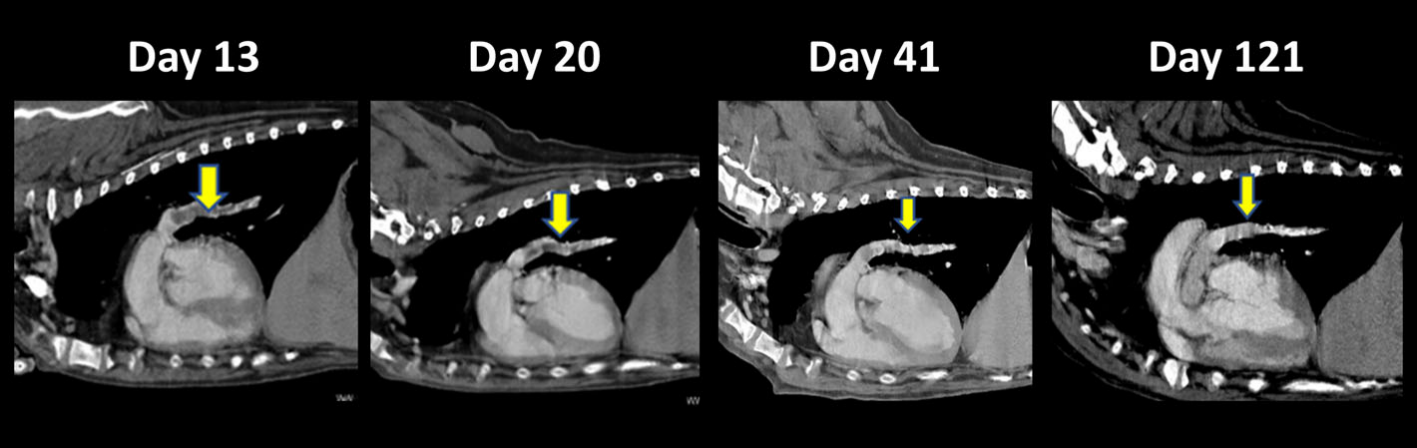
Contrast‐enhanced computed tomography scan without anesthesia on Days 13, 20, 41, and 121. After treating the pulmonary thrombosis, the contrast defects within the left and right pulmonary arteries (yellow arrows) gradually decreased.

On Day 10, The non‐sedated canine was manually restrained in right and left lateral recumbency under a flow‐by oxygen supply. Echocardiography showed that the right heart was more severely enlarged as compared with Day 1 (Figure 2 and Supplementary Table 1).^8^ No echocardiographic findings suggestive of congenital cardiac disease were observed. Additionally, left ventricle (LV) underfilling was observed based on the biplane area length method‐derived end‐diastolic LV volume.^7^, ^9^ Furthermore, we observed RV dysfunction based on decreased RV fractional area change normalized by body weight (RV FACn), B‐mode method‐derived tricuspid annular plane systolic excursion normalized by body weight (TAPSEn), and RV stroke volume measured using the cross‐sectional area method normalized to body surface area (RVSVI).^10^, ^11^ The TR velocity increased, and pulmonary vascular resistance estimated using echocardiography (PVRecho), calculated as (TR velocity [m/s])^2^/(velocity‐time integral of the pulmonary artery flow [cm]), was as high as 2.63.^12^ Additionally, we performed myocardial motion analysis using 2‐dimensional speckle‐tracking echocardiography (2D‐STE) as a sensitive indicator of myocardial function. We measured peak global strain in the LV longitudinal and circumferential directions (LV‐SL and LV‐SC, respectively) and in the longitudinal direction of the RV free wall (RV‐SL). The LV‐SL and LV‐SC were measured using the left apical 4‐chamber view and right parasternal short‐axis view at the level of the papillary muscle, respectively. The RV‐SL also was measured as the RV functional variable using the RV focus view.^13^ Specifically, RV‐SL was measured as segmental (basal, middle, and apical) and automatically generated global RV free‐wall strains. The analytical procedure and RI values for each strain were based on previous reports.^13^, ^14^ On Day 10, all of the strain values were below the RI.^11^, ^14^ In particular, the segmental RV‐SL indicated McConnell's sign, which is characterized by lowered basal and middle RV‐SL and preserved apical RV‐SL (Figure 3A and Supplementary Table 1) and is useful for detecting characteristics of acute pulmonary thrombosis in humans.^15^ We suspected that circulatory failure had worsened because of pulmonary thrombus PH. Therefore, PO BPS (15 μg/kg q12h) was initiated on Day 11, with expected pulmonary and systemic vasodilatory effects, protective effects on vascular endothelial cells, anti‐inflammatory effects, and antiplatelet effects. Additionally, after taking the dog safely from the oxygen cage, electrocardiography‐gained contrast‐enhanced computed tomography (CT; Canon Medical Systems Corporation, Tochigi, Japan) without anesthesia was performed to diagnose pulmonary thrombosis with certainty on Day 13. Contrast‐enhanced CT with 1.8 mL/kg contrast medium (iopamidol 300; Teva Takeda Pharma Ltd, Aichi, Japan) at a rate of 2.0 mL/s showed a wide range of contrast‐enhanced defects in the left and right pulmonary artery bifurcations at the periphery (Figure 4). Based on echocardiography on Day 10 and CT on Day 13, we diagnosed pulmonary thrombosis associated with PH. Therefore, monteplase additionally was administered IV on Days 14 and 18 (13 750 IU/kg) for thrombolysis.^16^ Benazepril (0.2 mg/kg PO q12h) was administered on Day 16 because of high systolic blood pressure (156‐162 mmHg).

On Day 20, the dog's respiratory status and exertional dyspnea improved markedly. The dog then was removed from the oxygen cage. Coagulation blood tests showed that FDP and d‐dimer concentrations were 9.3 and 6.0 μg/mL, respectively. The activated partial thromboplastin time was 13.6 s, and the fibrinogen concentration was 805 mg/dL. Echocardiography showed improvement in left ventricular underfilling based on increased end‐diastolic LV volume (Supplementary Table 1). Improvements in RV function, such as RV FACn, TAPSEn, and RVSVI also were observed. Furthermore, TR velocity and PVRecho decreased, respectively. Myocardial strains measured using 2D‐STE on Day 20 showed increases in LV‐SL, LV‐SC, and RV‐SL. The basal RV‐SL was increased, indicating improvement in McConnell signs (Figure 3B). The dog was discharged on Day 21. Oral medication included BPS (15 μg/kg q12h), clopidogrel (1.0 mg/kg q24h), benazepril (0.2 mg/kg q12h), rivaroxaban (0.5 mg/kg q24h; administered on Day 21). Rivaroxaban was administered instead of low‐molecular‐weight heparin.

Electrocardiography‐gained contrast‐enhanced CT showed that the thrombus size did not increase after Day 20 compared to Day 13. On Days 41 and 121, the peripheral pulmonary angiography area had expanded, indicating that the thrombus in the pulmonary artery had decreased in size (Figure 4). Additionally, ECG showed frequent ventricular premature complexes on Day 58. In human medicine, PH can cause myocardial ischemia, which might induce ventricular premature complexes.^16^ Therefore, although it was an off‐label use in veterinary medicine, isosorbide nitrate (1.2 mg/kg PO q12h), used in humans to treat ischemic heart disease, was added to improve coronary flow from Day 58. The patient has remained stable for 250 days. Respiratory state off of oxygen was stable, and exercise tolerance improved. Echocardiography on Day 250 showed further improvement in PH pathophysiology based on an increase in end‐diastolic LV volume and decrease in TR velocity and PVRecho (Supplementary Table 1). All myocardial strains were preserved until Day 250. Segmental RV‐SL did not show McConnell signs after starting medications for pulmonary thrombosis and PH. Up to 400 days, the dog has been progressing well with antithrombotic and pulmonary vasodilator treatment, including BPS.

## 3 DISCUSSION

In this dog, antithrombotic treatment, such as clopidogrel and low‐molecular‐weight heparin administration on Days 1‐10, was insufficient to improve hemodynamics and clinical signs, including severe dyspnea. In this case, administering monteplase (thrombolytic) and BPS (pulmonary vasodilator) improved the clinical signs and RV functional indicators, including PH pathophysiology. Although the improvements in this case might be mainly because of the chronic self‐fibrinolytic system, continuous antithrombotic therapy, and compensatory mechanisms of the RV (eg, RV hypertrophy, dilatation, and hyperfunction) and pulmonary artery (eg, increase in the effective pulmonary vascular bed) in the acute to chronic phase of PH, the thrombolytic effect of monteplase and the consequent decrease in pulmonary vascular resistance also provide additional benefits for improving pulmonary circulation.^17^ In addition, this dog had PH and slight systemic hypertension; therefore, the pulmonary and systemic vasodilatory effects of BPS also have provided additional benefits.^6^ Furthermore, although the dog was treated with clopidogrel and rivaroxaban, the protective effect on vascular endothelial cells and the antiplatelet effect of BPS might have prevented further thrombus development.^18^, ^19^, ^20^, ^21^ Future studies evaluating the anti‐thrombogenic effects of BPS in additional cases are expected to confirm the efficacy of BPS in dogs with pulmonary thrombosis.

The 2D‐STE‐derived segmental RV‐SL indicated McConnell's sign on Day 10, which is characterized by lowered basal and middle RV‐SL and preserved apical RV‐SL.^15^ As in human medicine, McConnell's sign, observed using 2D‐STE‐derived segmental RV‐SL analysis, was considered potentially useful for detecting acute pulmonary thrombosis in veterinary medicine.^17^ Furthermore, RV‐SL, not only global RV‐SL but also all segmental RV‐SL, markedly improved after monteplase and BPS administration. This outcome may be because thrombolytic treatments, such as clopidogrel, low‐molecular‐weight heparin, monteplase preparations, and BPS, decrease PVR by pulmonary vasodilatation. Additionally, RV and pulmonary vascular remodeling caused by chronic PH were considered. The LV‐SL and LV‐SC also improved after treatment, which may be related to an improvement in the myocardial function of the RV, increasing blood flow to the LV system. Therefore, the improvements in LV‐SL and LV‐SC may be a result of increased LV volume resulting from improved blood return to the LV. The higher PVRecho, in this case, decreased from Days 10 to 250 and was accompanied by improvement in clinical signs and RV functional indicators, such as RV‐SL. A previous study reported PVRecho as an indicator of the balance between the RV contractile and loading conditions in dogs with PH caused by left‐sided heart disease.^12^, ^22^ Therefore, PVRecho may be useful as an indicator of PVR and RV performance in patients with PH associated with causes other than left heart disease (precapillary PH) and also may be useful as a therapeutic monitoring index for PH.

Our case was managed for approximately 400 days by administration of monteplase and BPS in addition to the conventional treatment, including oxygen supplementation and antithrombotic drugs, which improved clinical signs associated with pulmonary thrombus PH. Thrombolytic treatment and addition of a pulmonary vasodilator should be considered if oxygen supplementation and antithrombotic therapy alone do not improve clinical signs associated with thrombus PH. Additionally, novel echocardiographic indicators, such as 2D‐STE‐derived segmental RV‐SL and PVRecho, might provide additional benefits for detecting and monitoring PH secondary to pulmonary thrombosis in dogs.

## CONFLICT OF INTEREST DECLARATION

The authors declare no conflicts of interest.

## OFF‐LABEL ANTIMICROBIAL DECLARATION

The authors declare no off‐label use of antimicrobials.

## INSTITUTIONAL ANIMAL CARE AND USE COMMITTEE (IACUC) OR OTHER APPROVAL DECLARATION

All procedures of this study followed the Guidelines for Institutional Laboratory Animal Care and Use of Nippon Veterinary and Life Science University in Japan. Written informed consent authorizing the use of data on the case was obtained from the owner.

## HUMAN ETHICS APPROVAL DECLARATION

The authors declare human ethics approval was not needed for this study.

## Supporting information


**Supplementary Table 1.** Course of echocardiographic variables in dog with pulmonary thrombosis.
