# Supplementary material for: Pulmonary thrombotic pulmonary hypertension managed using antithrombotic and pulmonary vasodilator treatment
Source: J Vet Intern Med. 2024 Apr 25;38(4):2333–8. doi: 10.1111/jvim.17089 (PMC11256183; doi:10.1111/jvim.17089)
Supplement: Supplementary file 1 — Supplementary Table 1. Course of echocardiographic variables in dog with pulmonary thrombosis. [file JVIM-38-2333-s001.pdf]

Supplementary Table 1. Course of echocardiographic variables in dog with pulmonary thrombosis.

Variables	Reference Interval	Day 0	Day 10	Day 20	Day 41	Day 250
End-diastolic LV volume (mL)	103.4 – 115.0[19]	82.8	77.8	85.3	98.3	107.7
EF (%)	49.8 – 53.1[19]	58.6	53.5	60.2	55.4	55.6
LA/Ao	1.2 – 1.3[19]	1.2	1.2	1.2	1.3	1.2
TR velocity (m/s)	Not applicable	3.4	4.4	3.9	3.3	3.2
End-diastolic RV area (cm^2/kg)	0.6 – 1.3[20]	1.4	1.6	1.4	1.3	1.4
End-systolic RV area (cm^2/kg)	0.3 – 0.8[20]	0.8	1.1	0.9	0.9	1.0
End-diastolic right atrial area (cm^2/kg)	0.45 – 0.76[20]	0.79	0.82	0.74	0.70	0.72
TAPSEn (mm/kg)	3.2 – 6.7[21]	3.7	3.6	5.4	5.2	5.3
RV FACn (%/kg)	46.3 – 76.9[22]	52.0	44.2	48.2	47.6	45.9
RV SVI (mL/m^2)	Not established	27.5	25.1	42.0	50.5	49.6
PVRecho	Not applicable	1.42	2.63	1.33	0.77	0.78

LV-SL (%)	14–23[10]	–	12.3	13.8	13.5	12.8
LV-SC (%)	19–25[10]	–	9.2	11.7	11.8	12.0
RV-SL (%)						
Global (%)	27.7 – 29.5[22]	–	12.7	17.3	19.0	17.4
Basal (%)	Not established	–	11.1	20.7	18.2	21.4
Middle (%)	Not established	–	14.4	16.8	16.7	17.0
Apical (%)	Not established	–	22.2	18.1	20.6	19.8

EF: ejection fraction; FACn: fractional area change normalized by body weight; LA/Ao: left atrial to aortic diameter ratio; LV-SC: left ventricular circumferential strain; LV-SL: left ventricular longitudinal strain; PVRecho: pulmonary vascular resistance estimated by echocardiography; RV: right ventricular; RV-SL: RV longitudinal strain; SVI: stroke volume normalized by body surface area; TAPSEn: tricuspid annular plane systolic excursion normalized by body weight; TR: tricuspid valve regurgitation.
